# Draft Genome Sequence of Bacillus anthracis N1, Isolated from a Recreational Freshwater Kettle Lake in Ontario, Canada

**DOI:** 10.1128/mra.01262-22

**Published:** 2023-03-13

**Authors:** Noah Bryan, Rebecca Anderson, Opeyemi U. Lawal, Valeria R. Parreira, Lawrence Goodridge

**Affiliations:** a Canadian Research Institute for Food Safety (CRIFS), University of Guelph, Guelph, Ontario, Canada; b Bayview Secondary School, Richmond Hill, Ontario, Canada; University of Delaware College of Engineering

## Abstract

Bacillus anthracis is widespread in soil and a causative agent of anthrax, primarily in herbivores. Here, we report the draft genome sequence of Bacillus anthracis strain N1, which was isolated from a recreational freshwater lake and found to carry multiple antibiotic resistance genes and biosynthetic gene clusters.

## ANNOUNCEMENT

Bacillus anthracis is a Gram-positive, facultatively anaerobic, spore-forming, rod-shaped bacterium and the causative agent of anthrax, which mainly affects livestock and wild animals (1–3). As part of a study assessing the bacterial diversity of Lake Wilcox, a freshwater kettle lake in Richmond Hill (Ontario, Canada; 43.9486°N, 79.4352°W), B. anthracis strain N1 was isolated and characterized. Briefly, freshwater samples (1 L) were collected from shallow regions of Lake Wilcox using a sterile bottle and kept at 4°C until processing. Water aliquots (1 mL) were serially diluted in 9 mL of modified saline-magnesium (SM) buffer (without gelatin) (4) and plated onto tryptic soy agar (TSA). The plates were incubated for 24 h at 37°C. Bacterial colonies of differing morphologies were subcultured on TSA. Genomic DNA was extracted from a pure colony using the DNeasy blood and tissue kit (Qiagen, Hilden, Germany) and sequenced using the Illumina DNA library prep tagmentation kit and IDT for Illumina DNA/RNA unique dual (UD) indexes as previously described (5).

A total of 1,843,542 sequencing reads for isolate N1 were obtained from an Illumina MiniSeq run using 150-bp paired-end sequencing. The raw reads were quality filtered using FastQC v0.11.9 (https://github.com/s-andrews/FastQC) and trimmed using Trimmomatic v0.39 (6). Reads with a Phred score of >20 were assembled *de novo* using SKESA v2.4.0 (7). The assembly quality was assessed using QUAST v5.2 (8). Genome annotation was performed using the NCBI Prokaryotic Genome Annotation Pipeline v6.3 (9). Putative antibiotic resistance genes were detected using CARD (10) and AMRFinder Plus v3.10.45 (11). The assembled genome was screened for plasmids using MOB-suite v3.1 (12). Biosynthetic gene clusters (BGCs) were assessed using antiSMASH v6 (13). Default parameters were used for all bioinformatics tools.

Isolate N1 was identified as Bacillus anthracis using *k*-mer-based species identification with the Kraken 2 database (14). B. anthracis N1 had a genome size of 5,347,760 bp, 50 contigs with an *N*_50_ value of 251,654 bp, 94× mean coverage, and 35.17% GC content. B. anthracis N1 contained 5,391 protein-coding sequences and 85 tRNAs. A comparative analysis between this strain and previously sequenced *Bacillus* spp. revealed 96.68% average nucleotide identity with B. anthracis CDC684 (GenBank accession number CP001215.1) using ANItools (15). Strain N1 carried genes on its chromosome coding for resistance to β-lactam (*bla*, *bla2*), glycopeptide (*vanRA*), macrolide (*mphL*), and streptothricin (*satA*). The MLST scheme for the Bacillus cereus group was applied to the strain and revealed two novel alleles (*glp-410*, *tpi-343*) and a new allele profile that was assigned as sequence type 2852 (ST2852) using PubMLST (16). B. anthracis N1 carried two plasmids, N1.p1 (contig N100023; 60,368 bp) and N1.p2 (contig N100026; 47,275 bp), with homology of 98% (coverage, 78%) to B. cereus BC07 pBC07-2 (CP072771.1), and 93.64% (coverage 68%) to Bacillus toyonensis BCT-7112 pBCT77 (CP006864.1), respectively. Nine regions encoding different BGCs were identified, including lasso peptide (contig N100003, position 339107 to 361279), terpene (contig N100005, position 147203 to 169056), and β-lactone (contig N100010, position 55309 to 80547), among others.

### Data availability.

This whole-genome shotgun project has been deposited at DDBJ/ENA/GenBank under the accession number JAPAEO000000000 and the SRA accession number SRR22013160 for the contigs and raw reads, respectively. The version described in this paper is version JAPAEO000000000.

## References

[1] Carlson CJ, Kracalik IT, Ross N, Alexander KA, Hugh-Jones ME, Fegan M, Elkin BT, Epp T, Shury TK, Zhang W, Bagirova M, Getz WM, Blackburn JK. 2019. The global distribution of Bacillus anthracis and associated anthrax risk to humans, livestock and wildlife. Nat Microbiol 4:1337–1343. doi:10.1038/s41564-019-0435-4. 31086311

[2] Norris MH, Kirpich A, Bluhm AP, Zincke D, Hadfield T, Ponciano JM, Blackburn JK. 2020. Convergent evolution of diverse Bacillus anthracis outbreak strains toward altered surface oligosaccharides that modulate anthrax pathogenesis. PLoS Biol 18:e3001052. doi:10.1371/journal.pbio.3001052. 33370274 PMC7793302

[3] Dale JL, Raynor MJ, Ty MC, Hadjifrangiskou M, Koehler TM. 2018. A dual role for the Bacillus anthracis master virulence regulator AtxA: control of sporulation and anthrax toxin production. Front Microbiol 9:482. doi:10.3389/fmicb.2018.00482. 29599764 PMC5862856

[4] Carey-Smith GV, Billington C, Cornelius AJ, Hudson JA, Heinemann JA. 2006. Isolation and characterization of bacteriophages infecting Salmonella spp. FEMS Microbiol Lett 258:182–186. doi:10.1111/j.1574-6968.2006.00217.x. 16640570

[5] Bryan N, Anderson R, Lawal OU, Parreira VR, Goodridge L. Draft genome sequence of Exiguobacterium sp. strain N5, isolated from a recreational freshwater kettle lake in Ontario. Microbiol Resour Announc, in press. doi:10.1128/mra.01261-22. PMC10112067 36880761

[6] Bolger AM, Lohse M, Usadel B. 2014. Trimmomatic: a flexible trimmer for Illumina sequence data. Bioinformatics 30:2114–2120. doi:10.1093/bioinformatics/btu170. 24695404 PMC4103590

[7] Souvorov A, Agarwala R, Lipman DJ. 2018. SKESA: strategic k-mer extension for scrupulous assemblies. Genome Biol 19:153. doi:10.1186/s13059-018-1540-z. 30286803 PMC6172800

[8] Gurevich A, Saveliev V, Vyahhi N, Tesler G. 2013. QUAST: quality assessment tool for genome assemblies. Bioinformatics 29:1072–1075. doi:10.1093/bioinformatics/btt086. 23422339 PMC3624806

[9] Tatusova T, DiCuccio M, Badretdin A, Chetvernin V, Nawrocki EP, Zaslavsky L, Lomsadze A, Pruitt KD, Borodovsky M, Ostell J. 2016. NCBI Prokaryotic Genome Annotation Pipeline. Nucleic Acids Res 44:6614–6624. doi:10.1093/nar/gkw569. 27342282 PMC5001611

[10] Alcock BP, Raphenya AR, Lau TTY, Tsang KK, Bouchard M, Edalatmand A, Huynh W, Nguyen A-LV, Cheng AA, Liu S, Min SY, Miroshnichenko A, Tran H-K, Werfalli RE, Nasir JA, Oloni M, Speicher DJ, Florescu A, Singh B, Faltyn M, Hernandez-Koutoucheva A, Sharma AN, Bordeleau E, Pawlowski AC, Zubyk HL, Dooley D, Griffiths E, Maguire F, Winsor GL, Beiko RG, Brinkman FSL, Hsiao WWL, Domselaar GV, McArthur AG. 2020. CARD 2020: antibiotic resistome surveillance with the Comprehensive Antibiotic Resistance Database. Nucleic Acids Res 48:D517–D525. doi:10.1093/nar/gkz935. 31665441 PMC7145624

[11] Feldgarden M, Brover V, Haft DH, Prasad AB, Slotta DJ, Tolstoy I, Tyson GH, Zhao S, Hsu C-H, McDermott PF, Tadesse DA, Morales C, Simmons M, Tillman G, Wasilenko J, Folster JP, Klimke W. 2019. Validating the AMRFinder Tool and resistance gene database by using antimicrobial resistance genotype-phenotype correlations in a collection of isolates. Antimicrob Agents Chemother 63:e00483-19. doi:10.1128/AAC.00483-19. 31427293 PMC6811410

[12] Robertson J, Nash JHE. 2018. MOB-suite: software tools for clustering, reconstruction and typing of plasmids from draft assemblies. Microb Genom 4:e000206. doi:10.1099/mgen.0.000206. 30052170 PMC6159552

[13] Blin K, Shaw S, Kloosterman AM, Charlop-Powers Z, van Wezel GP, Medema MH, Weber T. 2021. antiSMASH 6.0: improving cluster detection and comparison capabilities. Nucleic Acids Res 49:W29–W35. doi:10.1093/nar/gkab335. 33978755 PMC8262755

[14] Wood DE, Lu J, Langmead B. 2019. Improved metagenomic analysis with Kraken 2. Genome Biol 20:257. doi:10.1186/s13059-019-1891-0. 31779668 PMC6883579

[15] Han N, Qiang Y, Zhang W. 2016. ANItools Web: a Web tool for fast genome comparison within multiple bacterial strains. Database (Oxford) 2016:baw084. doi:10.1093/database/baw084. 27270714 PMC4911789

[16] Jolley KA, Maiden MCJ. 2010. BIGSdb: scalable analysis of bacterial genome variation at the population level. BMC Bioinformatics 11:595. doi:10.1186/1471-2105-11-595. 21143983 PMC3004885

